# Gene profiling of Toll-like receptor signalling pathways in neutrophils of patients with acute-on-chronic liver failure

**DOI:** 10.1186/s12967-021-03135-3

**Published:** 2021-11-13

**Authors:** Yi Zhang, Wei Wu, Yijie Wang, Lingjia Tong, Meng Hong, Qi Xia, Zhi Chen

**Affiliations:** 1 Department of Laboratory Medicine, The First Affiliated Hospital, College of Medicine, Zhejiang University, Hangzhou, 310003 China; 2 Key Laboratory of Clinical In Vitro Diagnostic Techniques of Zhejiang Province, Hangzhou, 310003 China; 3 State Key Laboratory for Diagnosis and Treatment of Infectious Diseases, Collaborative Innovation Center for Diagnosis and Treatment of Infectious Disease, The First Affiliated Hospital, Zhejiang University School of Medicine, Hangzhou, 310003 China; 4 Department of Laboratory Medicine, The Ningbo Ninth Hospital, Ningbo, 315000 China

**Keywords:** Toll-like receptors, mRNA, Pathogens and organ/tissue injury, Acute-on-chronic liver failure, Neutrophil

## Abstract

**Objectives:**

Toll-like receptors (TLRs) on neutrophils play a crucial role in detecting pathogens and organ/tissue injury in acute-on-chronic liver failure (ACLF). However, little is known about the exact mechanisms and potential signalling pathways. The aim of this study was to investigate alterations in TLR signalling pathways in neutrophils of ACLF patients.

**Methods:**

Twenty-seven patients with compensated cirrhosis (n = 9), decompensated cirrhosis (n = 9) and ACLF (n = 9) were enrolled in the study. Neutrophils were isolated, and alterations in TLR signalling pathways were evaluated using an RT^2^ Profiler™ PCR Array. The fold change for each gene (2(^−∆∆CT^)) was compared among the groups. Genes with a fold change ratio of ≥ 2 or ≤ 0.5 along with a *p* value of < 0.05 were considered to be differentially expressed.

**Results:**

A total of 17 genes were upregulated in neutrophils from patients with compensated cirrhosis and were mainly distributed in adaptors, TLR-interacting proteins and downstream pathways. Six genes were detected in patients with decompensated cirrhosis. A trend of downregulation of genes in the TLR signalling pathway was observed in neutrophils of patients with cirrhosis and ACLF. *TLR3*, *IFNG*, *IL1B*, *TBK1*, *CCL2* and *LTA* were downregulated in neutrophils. Moreover, CD14 and IL10 were upregulated in neutrophils of ACLF patients.

**Conclusions:**

TLR signalling pathway genes were differentially regulated in neutrophils between cirrhosis and ACLF. In ACLF patients, defective expression of TLR3 and IFN, along with enhanced CD14 and IL10 expression, was characterized by transcriptional alterations of neutrophils.

**Supplementary Information:**

The online version contains supplementary material available at 10.1186/s12967-021-03135-3.

## Introduction

Acute-on-chronic liver failure (ACLF) is a new clinical entity defined as acute exacerbation of underlying chronic liver disease, which usually triggers multiple organ failure and high short-term mortality [13]. The key driver of ACLF is the burst of systemic inflammation caused by bacterial infections, acute liver injury, variceal bleeding, or other acute injuries [3]. Specifically, the severity, duration and type of systemic inflammation are reported to substantially affect the outcome of ACLF [6, 7]. To the best of our knowledge, the function of neutrophils is critical in determining the nature of systemic inflammation [27]. On this basis, it is reasonable to assume that functional modulation of the innate immune system may serve as a potential treatment option for ACLF.

Toll-like receptors (TLRs), probably the most important class of pattern-recognition receptors, are crucial in the regulation of the innate immune system [18]. They were first identified as key receptors in the recognition of pathogen-associated molecular patterns (PAMPs). Currently, 11 types of TLRs have been identified in humans, among which 10 are functional [18]. For example, in combination with lipopolysaccharide-binding protein and CD14, TLR4 on the surface of innate immune cells can recognize lipopolysaccharide (LPS), which is a component present in most gram-negative bacteria [21]. In addition, TLR3 located on endosomes can recognize retroviral double-stranded RNA [2]. Recently, TLRs have been shown to be involved in sterile inflammation caused by tissue damage [29]. TLRs are capable of recognizing damage-associated pattern molecules (DAMPs), which subsequently activate innate immune cells [29]. For instance, cellular HMGB1, a chromatin protein, can be recognized by TLR4 on neutrophils, leading to the formation of neutrophil extracellular traps (NETs) [14].

PAMPs and DAMPs have been detected in the serum or plasma samples of ACLF patients [9, 19]. Increased TLR expression and altered neutrophil responses were reported in ACLF patients [5, 26]. However, there is still a lack of a comprehensive understanding of TLR signalling cascades. In this study, RNA qRT-PCR array technology was utilized to evaluate the relevant expression of TLR-regulated genes in neutrophils from ACLF patients. We aimed to identify the key TLR pathways that may serve as therapeutic targets for treating ACLF.

## Materials and methods

### Patients

A total of 27 patients with compensated cirrhosis (n = 9), stable cirrhosis (n = 9) and ACLF (n = 9) admitted to the First Affiliated Hospital of Zhejiang University were enrolled in this study. Meanwhile, 9 matched healthy volunteers who received physical examinations during the same period served as healthy controls (HCs). Those diagnosed with ACLF based on the EASL-ACLF criteria [20] were included in this study. Patients with the following conditions were excluded from the study: those with human immunodeficiency virus infection; confirmed or suspected malignancies; pregnant women; received organ transplantation previously; received administration of immunosuppressive drugs; or those with active bacterial infection. Written consent was obtained from each patient or their legal relations. The study protocols were approved by the Ethics Committee of the First Affiliated Hospital of Zhejiang University and complied with the Helsinki Declaration.

### Isolation of neutrophils

Peripheral venous blood samples were collected within 24 h upon admission. Neutrophils were isolated from whole blood samples using a Polymorph prep kit (Axis-Shield PoC AS, Norway) according to the manufacturer’s instructions. Cells were resuspended in 1 ml TRIzol reagent (Invitrogen, CA, USA), followed by storage in liquid nitrogen.

### RNA extraction, cDNA and PCR array

An RT^2^ Profiler™ PCR Array for Human TLR Signalling Pathway (PAHS-018Z, QIAGEN) was used in this section. RNA extraction, cDNA synthesis and PCR array preparation were performed according to the manufacturer’s instructions. Briefly, the experimental RNA samples were converted into first-strand cDNA using an RT^2^ first-strand kit (QIAGEN). Afterwards, the cDNA was mixed with an appropriate RT^2^ SYBR Green Mastermix (QIAGEN). The mixture was aliquoted into the wells of an RT^2^ Profiler PCR Array. The amplification results were expressed as Ct and normalized to the housekeeping gene 18S rRNA (^∆CT^). The fold change of gene expression was calculated as 2(^−∆∆CT^).

### TLR signalling pathway mapping

Kyoto Encyclopedia of Genes and Genomes (KEGG) Mapper was used to analyse differentially expressed genes^16^. The ratio mean of gene expression was calculated in the control and ACLF groups, which was visualized in a colour code in the TLR signalling pathway (KEGG hsa04620).

### Statistical analysis

All data were analysed using SPSS 16.0 software (SPSS, Chicago, IL) and R 4.0.3. Student’s t-test, Mann–Whitney U-test or Chi square test was utilized for the data comparison among groups. A two-sided P value of < 0.05 was considered to be statistically significant. PCA was performed by ggbiplot R package. Volcano plots were plotted by ggplot2 R package.

## Results

### Patient’ characteristics

As shown in Table 1, age and sex were matched among the HC, compensated cirrhosis and stable cirrhosis groups; however, they were not matched among the ACLF patients. As expected, compared to other groups, ACLF patients had severe hepatic dysfunction, jaundice and coagulopathy, as evidenced by laboratory measures and scores of disease severity.

**Table 1:** Demographic and clinical characteristics of the study participants

Variables	Health controls (n = 9)	Compensated cirrhosis (n = 9)	De-compensated cirrhosis (n = 9)	ACLF (n = 9)
Male/female	6/3	5/4	6/3	6/3
Age	53.4 ± 3.7	54.4 ± 6.7	58.3 ± 12.4	47.3 ± 10.9
Aetiologies of cirrhosis				
HBV	0	7	6	8
Non-HBV	9	2	3	1
De-compensation events				
Ascites	–	0	6	2
UGH	–	0	3	1
HE	–	0	0	0
Jaundice	–	0	0	9
Organ failure	–			
Liver failure	–	0	0	9
Coagulation failure	–	0	0	6
Kidney failure	–	0	0	0
Cerebral failure	–	0	0	0
Circulation failure	–	0	0	0
Lung failure	–	0	0	0
WBC (10^9^/L)	5.4 (3.5)	4.2 (2.8)	2.1 (2.8)	7.4 (7.1)
PLT (10^9^/L)	123 (24)	89 (111)	60.5 (57)	109 (58)
TB (umol/L)	6 (3.4)	14 (15.5)	19.5 (19.5)	368 (135.3)
INR	1 (0.1)	1.1 (0.1)	1.4 (0.5)	2.6 (0.9)
Creatinine(umol/l)	78 (22)	64 (20)	68 (12)	66 (30.8)
Albumin(g/L)	43 (5.7)	40.3 (11.2)	33 (9.8)	31.7 (5.6)
MELD score	–	5.3 (7.4)	8.5 (7)	25 (5)
CLIF-C ACLFs	–	–	–	41.5 (8.3)

*HBV* hepatitis B virus, *UGH* upper gastrointestinal haemorrhage, *HE* hepatic encephalopathy, *Jaundice* TBil ≥ 85 µmol/L, *WBC* white blood cell, *PLT* platelet, *TB* total bilirubin, *INR*: international normalized ratio, *MELD* model for end-stage liver disease,*CLIF*-*C* *ACLFs* Chronic liver failure Consortium Acute-on-chronic liver failure scores

### PCR array in polymorphonuclear neutrophils (PMNs)

**Fig. 1:**
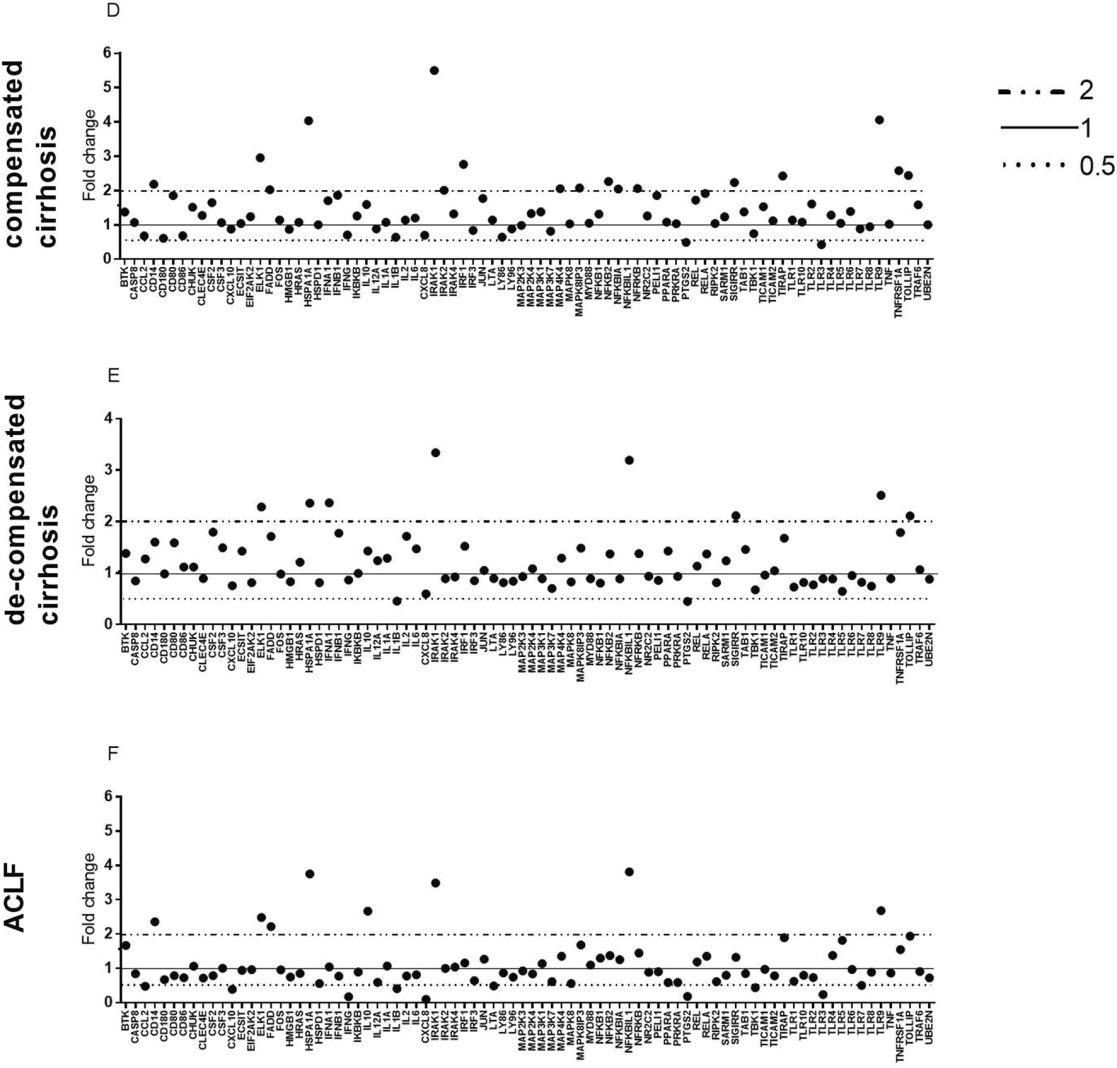
Fold change ratios of TLR signalling pathway-related genes of PMNs in compensated cirrhosis, decompensated cirrhosis and ACLF in comparison to healthy controls (HCs). Full line indicated reference level of HC. The dotted line indicates a fold change ratio of ≤ 0.5 compared with the reference level. Half-full and half-dotted lines demonstrate a fold change ratio of ≥ 2 in comparison to the reference level

**Fig. 2:**
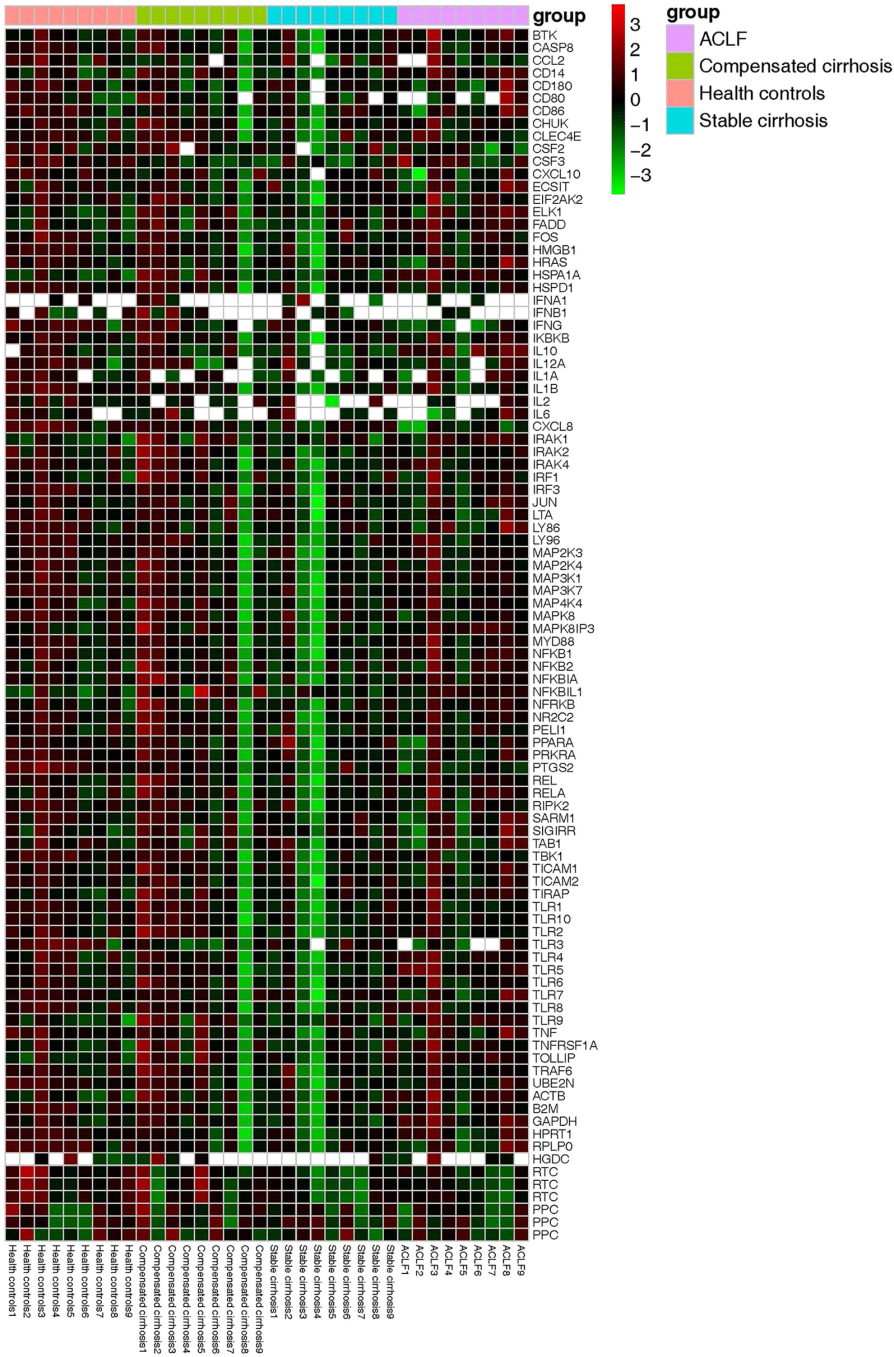
Individual TLR signalling pathway-related gene expression of PMNs among the HC, compensated cirrhosis, decompensated cirrhosis and ACLF groups. The relative expression level is indicated by the colour code. Genes with significant upregulation are coloured red, and those with significant downregulation are marked green. The ubiquitous expression level was shown in black

**Fig. 3:**
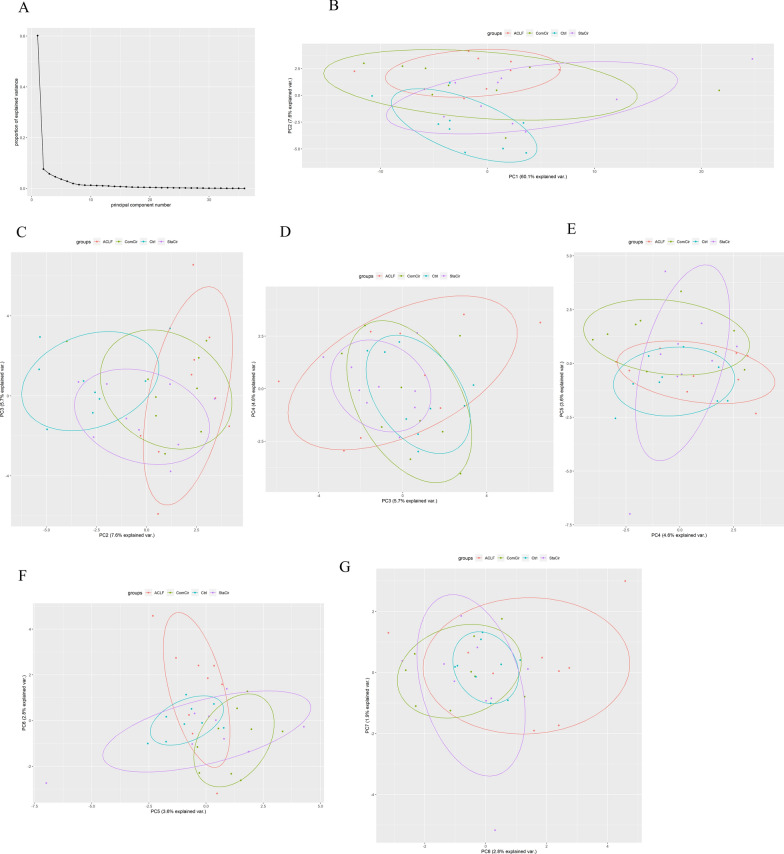
Results of PCA. **A** Screeplot showing that the first 7 principal components mainly influenced data attribution. **B**–**G** showed clustering condition on different PCs. ACLF group showed significant difference in these PCs

Genes of the TLR signalling pathway were differentially expressed in PMNs from compensated cirrhosis to ACLF patients compared to HCs. Patients with compensated cirrhosis represented a predominant upregulation of TLR pathway-related genes, including *NFKBIA*, *NFΚB*, *NFRKB*, *NFΚBIL1*, *MAP4K4*, *TNFRSF1A*, *IRF1* and *ELK1*, which were distributed in downstream pathways and target genes (i.e., *MAPK8IP3*, *TIRAP*, *CD14*, *TOLLIP* and *HSPA1A*) among adaptors and TLR-interacting protein genes, SIGIRR and TLR9 among *TLR* genes, and FADD and IRAK1 among the effector genes. Only *TLR3* and *PTGS2* were downregulated. Patients with decompensated cirrhosis presented a more limited alteration in TLR signalling genes, including 6 of the 17 upregulated genes observed in compensated cirrhosis, SIGIRR, IRAK1, HSPA1A, TOLLIP and ELK1, as well as downregulation of *IL-1B*. In contrast, ACLF patients had a different type of alteration in TLR signalling genes, which was characterized by downregulation of *TLR3*, *CXCL8*, *IFNG*, *IL1B*, *TBK1*, *CCL2* and *LTA*. In addition to FADD, IRAK1, CD14, HSPA1A, NFKBIL1 and ELK1 were commonly upregulated in cirrhosis; however, IL-10 was uniquely upregulated in ACLF (Table 2). The gene expression profiles in the different groups are listed in Fig. 1. Individual gene expression profiles are listed in Fig. 2. PCA showed clustering of all the samples, which was consistent with the original groupings. The results of PCA are shown in Fig. 3. Volcano plots were listed in Additional file 1: Fig. S1.

**Table 2:** List of differentially expressed genes of TLR signalling pathways between patients and healthy controls

Gene symbol	Gene	Neutrophils		
		C-LC	D-LC	ACLF
		Fold change P value	Fold change P value	Fold change P value
Toll like receptor	*TLR10*	–	–	–
	*TLR3*	0.4231 0.0478	–	0.2398 0.0016
	*TLR9*	4.0564 0.0009	2.5115 0.03556	2.6805 0.023
	*SIGIRR*	2.2314 0.0105	2.1148 0.0447	–
Effectors	*FADD*	2.022 0.0302	–	2.2185 0.0133
	*IRAK1*	5.4967 0.0003	3.3386 0.0193	3.4884 0.0089
	*IRAK2*	–	–	–
Interacting proteins and adaptors	*CD14*	2.1815 0.0116	–	2.3584 0.0033
	*HSPA1A*	4.0346 0.0017	2.3591 0.0183	3.7515 0.0039
	*MAPK8IP3*	2.0727 0.0129	–	–
	*TOLLIP*	2.4367 0.0113	2.111 0.0039	–
	*TIRAP*	2.4229 0.0012	–	–
Regulation of adaptive immunity	*IFNG*	–	–	0.173 0.0006
	*IL10*	–	–	2.6697 0.0249
	*IL12A*	–	–	–
	*IL1B*	–	0.4534 0.0017	0.4067 0.0025
	*IL2*	–	–	–
Downstream pathway of toll like receptors				
NFKB signalling	*FADD*	2.022 0.0302	–	2.2185 0.0133
	*IL10*	–	–	2.6697 0.0249
	*IL1B*	–	0.4534 0.0017	0.4067 0.0025
	*IRAK1*	5.4967 0.0003	3.3386 0.0193	3.4884 0.0089
	*IRAK2*	–	–	–
	*MAP4K4*	2.0512 0.0018	–	–
	*NFKB2*	2.2645 0.0060	–	–
	*NFKBIA*	2.0441 0.0170	–	–
	*NFRKB*	2.056 0.0292	–	–
	*TNFRSF1A*	2.5753 0.0015	–	–
	*CXCL8*	–	–	0.0959 0.0422
	*LTA*	–	–	0.489 0.0157
JNK/p38 signalling	*ELK1*	2.9532 0.0012	2.2857 0.0128	2.4822 0.0054
	*IL1B*	–	0.4534 0.0017	0.4067 0.0025
	*MAPK8IP3*	2.0727 0.0129	–	–
JAK/STATsignalling	*CCL2*	–	–	–
	*IFNG*	–	–	0.173 0.0006
	*IL2*	–	–	–
Interferon regulatory factor signalling	*CXCL10*	–	–	–
	*IFNB1*	–	–	–
	*IFNG*	–	–	0.173 0.0006
	*IRF1*	2.7619 0.0012	–	–
	*TBK1*	–	–	0.4399 0.0013
Cytokine signalling	*CCL2*	–	–	–
	*IL1B*	–	0.4534 0.0016	0.4067 0.0025
	*IRAK1*	5.4967 0.0003	3.3386 0.0193	3.4884 0.0089
	*IRAK2*	–	–	–
	*SIGIRR*	2.2314 0.0105	2.1148 0.0045	–
	*TNFRSF1A*	2.5753 0.0015	–	–
NF/IL6 pathway	*PTGS2*	0.4895 0.0038	–	0.1811 0.0002

*C-LC* compensated liver cirrhosis, *D-LC* decompensated liver cirrhosis, *ACLF* acute-on-chronic liver failure

### TLR signalling pathway mapping

**Fig. 4:**
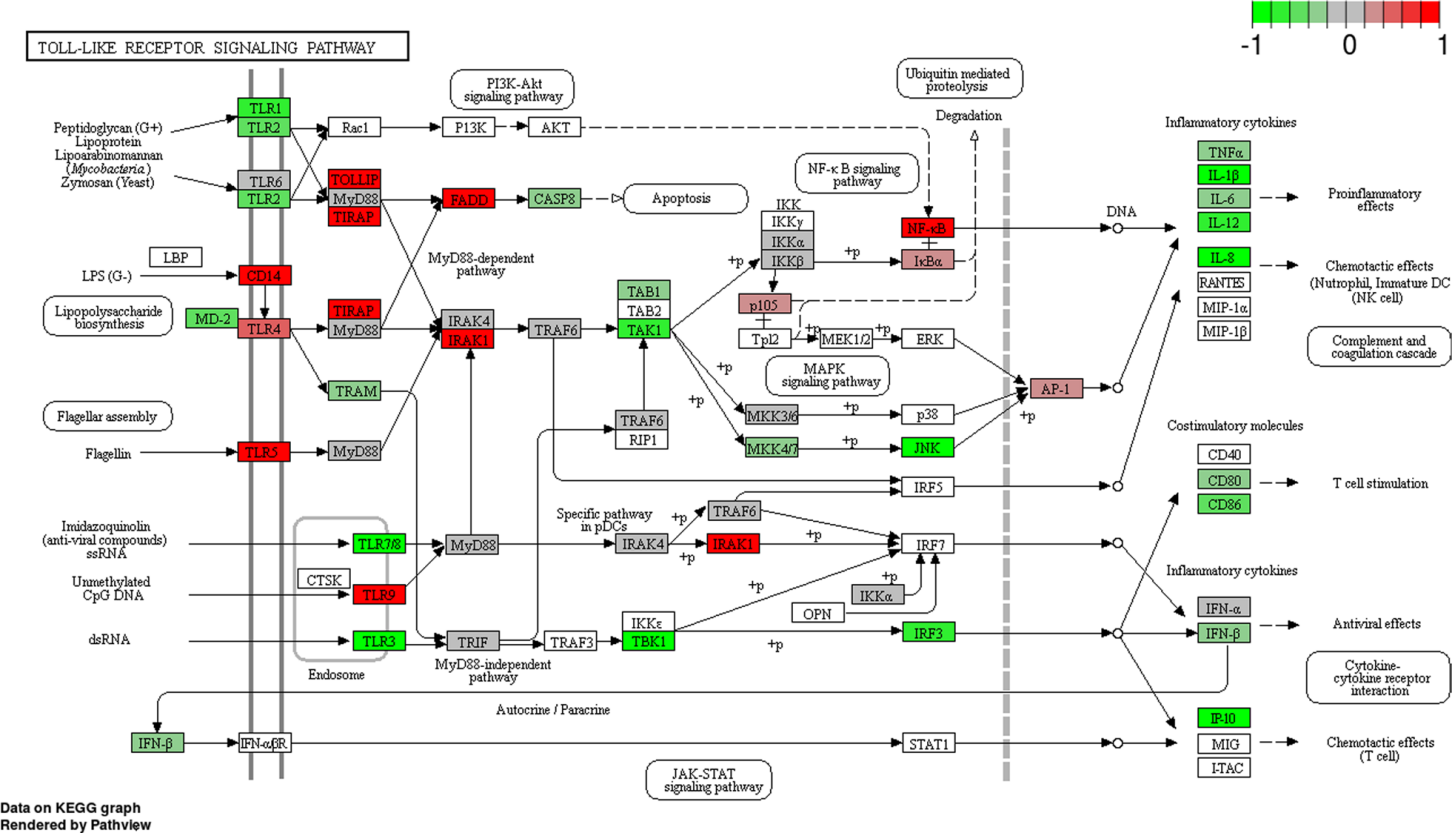
Mapping of TLR signalling pathway in ACLF patients. Each gene in the KEGG TLR signalling network (KEGG hsa04620) was coloured according to its expression level in PMNs of the ACLF group compared with that of HCs. Genes that were significantly upregulated are coloured red, and those that were significantly downregulated are marked green. The ubiquitous expression level is indicated in grey. The gene that was shown in the blank was not measured in the experiment

In this section, we explored the specific TLR pathway involved in the pathogenesis of ACLF. The KEGG TLR signalling network (KEGG hsa04620) was utilized to compare the expression levels of the HC and ACLF groups (Fig. 4). TLR signalling pathway mapping showed that the TLR3 pathway was significantly downregulated in neutrophils from ACLF patients.

## Discussion

TLRs are transmembrane proteins expressed in innate immune cells, especially neutrophils, and play a central role in detecting invading microbes or endogenous danger signals and initiating corresponding immune responses [18]. Ten TLRs (i.e., TLR1-TLR10) have been identified in humans, while different TLRs can recognize various ligands, including bacterial cell wall components and viral double-stranded RNA [28]. There were five housekeeping genes and 84 genes related to TLR-mediated signal transduction. These genes were divided into different groups according to their functions: TLR, adaptor and interacting proteins, effectors, regulation of the adaptive immunity group, downstream pathways and target genes. In this study, we found a stepwise downregulation of TLR3 in neutrophils from patients with cirrhosis to those with ACLF. These results were consistent with a recent study showing defective TLR3 expression in ACLF patients [4]. As previously described, TLR3 could recognize retroviral dsRNA, which then triggered the activation of the transcription factor interferon regulatory factor-3 (IRF3) in a MyD88-independent manner. This allowed the induction of IFNs [8]. Thus, defective TLR3 signalling may lead to downregulation of type I and II interferons, interferon regulatory factor TBK1 and interferon-stimulated gene (ISG) *CXCL10* expression in immune cells of ACLF patients. Although TLR4 engagement also induced IFNs in immune cells [10], TLR4 signalling was not suppressed in ACLF patients. Therefore, defective TLR3 signalling may be a major contributor to the downregulation of interferons and their ISGs in ACLF patients. Along with the IFNs, genes regulating adaptive immunity, including IL-2 and IL-12A, were significantly downregulated. It has been shown that enhanced activity of type I IFN signalling pathways is associated with excessive inflammation and tissue damage in mouse models of endotoxin shock [17] and autoimmune diseases [22]. Thus, downregulation of type I interferon regulatory signalling may be protective against excessive inflammation in ACLF patients. In parallel, the anti-inflammatory cytokine IL-10 was significantly upregulated in neutrophils, and pro-inflammatory IL-1B was downregulated specifically in neutrophils of patients with ACLF. Taken together, these findings suggested that peripheral neutrophils in ACLF patients showed defective expression of TLR3 and IFN, together with upregulation of the anti-inflammatory cytokine IL-10 at the transcriptional level.

Downregulation of IFNG was observed in all three groups, although it was not significant in the C-LC and D-LC groups, which also indicated that in chronic hepatic diseases, the level of immunoregulation was decreasing. IFN- reduces IL-4 production in TH1 cells [16] and plays a significant role in the class I antigen presentation pathway [24]. These results all indicated that during the development of chronic HBV-hepatitis cirrhosis, the level of immunoreaction decreased.

The TLR signalling transcriptional landscape of immune cells in ACLF was almost completely distinct from that in cirrhosis patients, which supported the clinical and experimental data that ACLF patients showed different immune dysfunctions [1]. Transcriptional alterations in immune cells may have significant clinical implications. The prevailing theory hypothesized a shift from systemic inflammatory response syndrome (SIRS) to compensatory anti-inflammatory response syndrome (CARS) during the natural history of ACLF, which led to immune paralysis and increased susceptibility to infections [15]. Indeed, several clinical studies have confirmed that ACLF patients show a higher incidence of bacterial infection than their non-ACLF counterparts [11, 23]. In human and animal studies, there was a link between impaired cellular immune function and an increased risk of bacterial infection, loss of infection control and infection-associated mortality [4, 12, 25]. Our findings revealed that a reduction in TLR signalling may impair TLR-driven neutrophil responses, which therefore represents a potential therapeutic target. A recent study demonstrated that the TLR-3 agonist poly(I:C) effectively reconstituted innate immune function in ACLF, which may serve as a promising TLR-based immunotherapy for ACLF [4].

However, there are some limitations in this study. First, there is a necessity for further verification of the PCR array results due to the limited population consistency and sample amount. Systematic studies in larger sample pools with multiple population features should be performed to provide more robust evidence. Second, considering changes in transcription and translation processes, the expression of genes could not represent the actual biological response. Thus, in vitro and animal studies are required to investigate the feasibility of crucial roles in the TLR3 signalling pathway that may serve as a target for the treatment of ACLF. Additionally, statistics are limited. There was a lack of multiple corrections of p values in the analysis of PCR array data. These results indicate that the study needs to be repeated and confirmed in studies with larger sample pools and through longer time periods.

In summary, genes in the TLR signalling pathway were differentially expressed in neutrophils through different stages of cirrhosis. In addition, we identified a dysregulated key TLR pathway in the neutrophils of ACLF patients. In the future, further investigation is required to test its potential as a therapeutic target.

## Supplementary Information

**Additional file 1: Figure S1.** Volcano plots showed upregulation and downregulation of marker genes compared to the healthy control group. A. Compensated cirrhosis group compared to the healthy control. B. Decompensated cirrhosis group compared to the healthy control group. C. ACLF group compared to the health control.

## Data Availability

The datasets used and/or analysed during the current study are available from the corresponding author on reasonable request.

## Abbreviations

TLR: Toll-like receptor
ACLF: Acute-on-chronic liver failure
PMN: Polymorphonuclear neutrophil
DAMPs: Damage-associated pattern molecules
HC: Healthy control
C-LC: Compensated liver cirrhosis
D-LC: Decompensated liver cirrhosis
