# Supplementary figures and images for: Gene profiling of Toll-like receptor signalling pathways in neutrophils of patients with acute-on-chronic liver failure
Source: J Transl Med. 2021 Nov 13;19:465. doi: 10.1186/s12967-021-03135-3 (PMC8590218; doi:10.1186/s12967-021-03135-3)

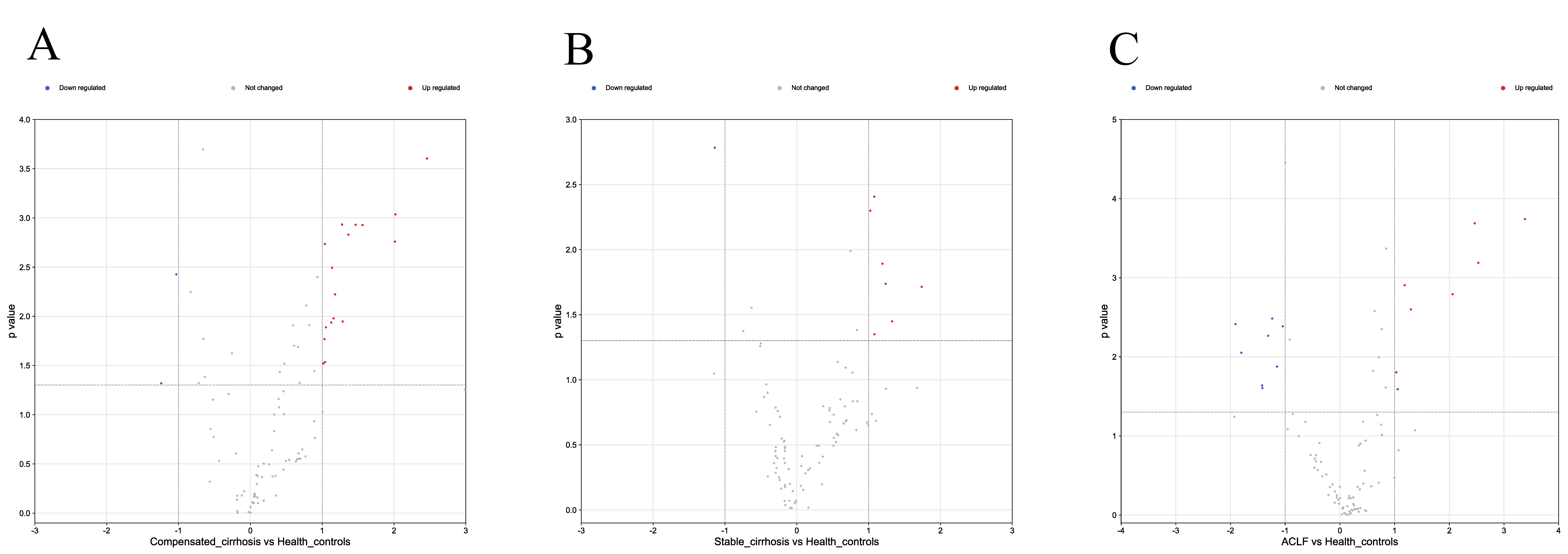

Supplement: Supplementary file 1 — Additional file 1: Figure S1. Volcano plots showed upregulation and downregulation of marker genes compared to the healthy control group. A. Compensated cirrhosis group compared to the healthy control. B. Decompensated cirrhosis group compared to the healthy control group. C. ACLF group compared to the health control. [file 12967_2021_3135_MOESM1_ESM.png]
